# Magnetic resonance evaluation of knee osteoarthritis among the Saudi Population

**DOI:** 10.12669/pjms.35.6.874

**Published:** 2019

**Authors:** Majed Gorayan Alrowaili

**Affiliations:** Dr. Majed Gorayan Alrowaili, Department of Surgery (Orthopedic Division), Faculty of Medicine, Northern Border University, Arar, Saudi Arabia

**Keywords:** Arar, Degenerative disorders, Knee osteoarthritis, Magnetic resonance imaging (MRI), Young population, KOA: Knee osteoarthritis, MRI: Magnetic resonance imaging, OA: Osteoarthritis

## Abstract

**Background and Objectives:**

Osteoarthritis (OA) is the most prevalent worldwide joint degenerative disorder with high morbidities and disabilities. The current study aimed to investigate the prevalence of knee osteoarthritis (KOA) in Arar by using magnetic resonance imaging (MRI).

**Methods:**

The prevalence of KOA was studied in Arar through MRI evaluation of randomly chosen sample from patients and their relatives attending the Prince Abdul Aziz Bin Mussad Hospital from October 2015 to November 2016.

**Results:**

A total of 410 participants were enrolled in the study [328 (80%) male and 82 (20%) females]. After MRI, 163 participants [39.75% (95% CI) = 35.14 - 44.57%)] were diagnosed with KOA. The prevalence of OA was about 25.6% (95% CI = 20.8 - 31.1%) below the age of 40 years, which was found to increase by age in the enrolled volunteers. KOA prevalence was higher in females than males (75.6% and 27.7% respectively). There was a significant association between the age and genders of the participants and the prevalence of OA (p-value < 0.0001 for both variables). There was also a significant association between the age and gender of the participants and the MRI-estimated grading (p-value < 0.0001 and 0.0044 respectively).

**Conclusion:**

KOA is a common disease among Arar young population, especially females. Its prevalence increases by age with higher grades of severity affecting the elderly.

## INTRODUCTION

Osteoarthritis (OA) is the most prevalent degenerative disorder of the joint around the globe.^1^ It is a major cause of significant disability and morbidity. Osteoarthritis carries considerable social and economic impact personally and in terms of public health. Due to the high morbidity burden of OA, a huge direct and indirect annual cost is incurred, estimated to be over 40 billion dollars in the USA alone.^2^ The prevalence of OA varies in different parts of the world from 3.8 - 70% depending on the methodologies used for its diagnosis and the studied populations.^3^,^4^

Osteoarthritis is a multifactorial heterogeneous human health issue due to an intrinsic problem in the articular cartilage resulting from repeated mechanical and biochemical stressors leading to the breakdown of the extracellular matrix and the impairment of repair. The risk factors attributed to the development of OA include advanced age, sex, trauma, obesity, genetics, diabetes mellitus, malalignment and several dietary factors such as low levels of vitamin D, C, and K.^2^-^6^

Any joint may be affected by OA but the most prevalent sites involved in OA include knee joint, hip joint and joints of hand.^7^,^8^ Diffuse knee pain is the most prevalent symptom of knee osteoarthritis (KOA).^9^ This pain is an important causative factor of disability among patients with OA.^10^ The pain limits joint activity, which may contribute to the development of atrophic changes in the surrounding muscles.^11^ The other important clinical features of KOA are tenderness, stiffness, and locking.

The radiological evaluation of the affected joints is of paramount importance in the diagnosis of OA. Radiologically, three important grading scales (Kellgren–Lawrence, Ahlback, and Brandt) are frequently employed for the diagnosis and grading assessment of OA.^12^ Kellgren–Lawrence (KL) is the most frequently-used scale and has been recommended by the World Health Organization (WHO).^13^ However, there are limitations to the KL scale as it is defective for characterization of cartilage loss and identification of bone marrow changes in KOA.

Recently, magnetic resonance imaging (MRI)-based assessment of KOA has become a well-established and reliable tool for assessment of OA cases. MRI-based morphologic scoring has been applied in cross-sectional, epidemiological, and clinical studies. MRI is more appropriate to evaluate the cases of KOA when compared to the traditional X-ray photographs as it allows the meticulous study of knees’ structures including; cartilage, articular bones, osteophytes, and the joint soft tissues structures as ligaments, menisci, synovial membrane, and bursa with better assessment and grading of KOA relative to traditional methods.^14^-^16^

In the current study, MRI was used, for the first time in Saudi Arabia, to assess the prevalence of OA among the adult population in Arar in comparison with elderly. In addition, MRI was used to assess the grading of OA in the MRI-confirmed KOA cases.

## METHODS

The present study evaluated a randomly-selected population of patients and their relatives from people attended to Prince Abdul Aziz Bin Mussad Hospital, Arar, Saudi Arabia from October 2015 to November 2016.

### Ethical considerations

The current study design was approved by the Local Bio-ethical Committee of Northern Border University (Certificate of Ethical Approval #156/39/49/D). Informed consent documents were prepared and approved by the committee and signed by the participants after receiving an explanation of the methods and objectives of the study. Confidentiality was maintained in all steps of the research project and all procedures were performed in accordance with the Declaration of Helsinki.

All participants who submitted informed consent documents and were devoid of any exclusion criteria were enrolled in the study. The exclusion criteria included a history or manifestation of septic arthritis, rheumatoid arthritis, road-side accidents, previous knee surgery, and participants who were unable to undergo MRI of the knee. Participants provided complete medical histories and underwent clinical examination prior to MRI evaluation.

MRI examination was performed with a 1.5-T MRI instrument (GE Healthcare, CITY, STATE, USA) using a quadrature receiver knee coil for signal reception. Due to the relatively long acquisition protocol, patients were immobilized using hook-and-loop straps. MRI sequences of axial, sagittal, and coronal planes were performed following Joshi et al. (2008).^17^ Slices were obtained at 4 - 5 mm thickness with an interscan gap of 0.5 - 1m. All compartments of the knee joint were assessed, including the articulating surfaces of the femur and tibia, the articulating facets of the patella, and the trochlea of femur. For the grading of MRIs, Park et al. (2013)^18^ grading system was used depending on cartilage defect, bone marrow edema, osteophytes, and bony ulceration (Table-I). Cartilage defect in the grading was based on Noyes classification system.^19^ The most severely affected area was marked for grading purposes.

**Table I:** Park MRI scoring system of osteoarthritis.

Grade 0	No cartilage injury with no osteophytes or osteophytes <5mm
Grade I	Altered internal signal of the cartilage and at least one of the followings: Osteophytes >5mm, Bone marrow edema >10mm, subchondral cyst>10mm.
Grade II	: Cartilage defect less than 99% and at least one of the followings: Osteophytes >5mm, Bone marrow edema >10mm, subchondral cyst>10mm.
Grade III	Total Cartilage defect and at least one of the followings: Osteophytes >5mm, Bone marrow edema >10mm, subchondral cyst>10mm.
Grade IV	Total cartilage loss with meniscal injury grade III.

The MRIs for each participant were assessed independently by two competent radiologists. All these MRIs data and relevant clinical details were recorded and subsequently analyzed.

### Statistical analysis

Prevalence of OA as the cause of knee pain was calculated by dividing the number of cases diagnosed as KOA by the total number of cases enrolled in the study. The 95% confidence intervals (CIs) were calculated according to Newcomb (1998).^20^ Chi-square test was used for association statistics. The statistical analyses were conducted in Prism5 (Graph Pad Software Inc., San Diego, CA, USA). P-values below 0.05 were considered as statistically significant.

## RESULTS

During the study period, a total of 410 participants were included in the study. They included 328 (80%) male and 82 (20%) female patients with an average age of 34.6 ± 12.4 years (range: 17 - 85 years).

**Fig. 1:**
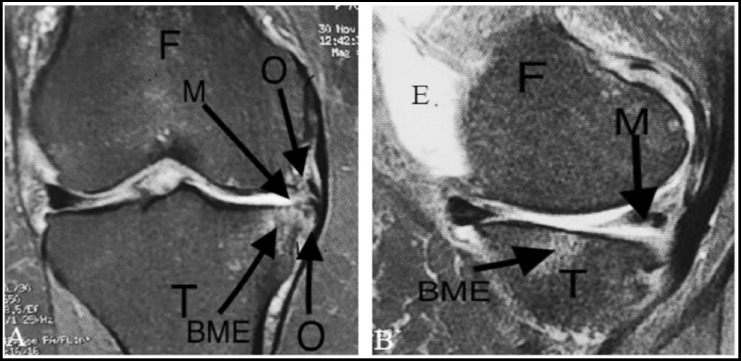
Representative figure for the knee osteoarthritis (KOA) lesions documented by Magnetic resonance imaging (MRI) of the studied population [a- coronal, b- sagittal]. BME, Bone marrow edema; E, Effusion; F, femur; M, Meniscus (post horn medial meniscus) tear; O, osteophytes; T, Tibia..

**Fig. 2:**
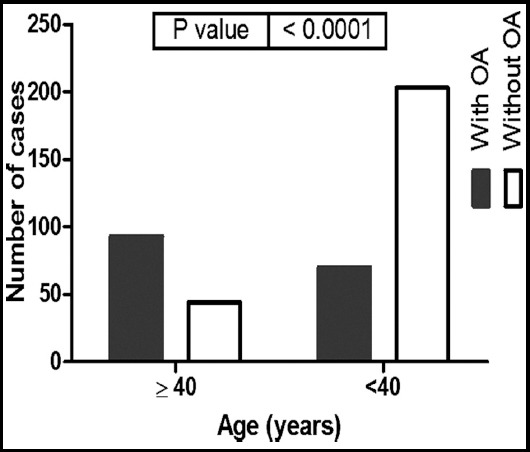
Age distribution of KOA cases among the study population. P-values were estimated by chi-square test.

Data from the MRI and Park’s scoring system are represented in Fig. 1. Among the 410 volunteers, 163 participants were diagnosed with KOA by MRI with a general prevalence of 39.75% (95% CI = 35.14 - 44.57%). For patients below 40 years of age, the prevalence of OA was about 25.6% (95% CI = 20.8 - 31.1%), which increased to 67.8% (95% CI = 59.7 - 75.1%) among participants above the age of 40; therefore, the relative risk of aging as a risk factor was 2.6 (95% CI = 2.1 - 3.3) (Fig. 2). Interestingly, MRI showed KOA in 30 (7.3%) participants (7 cases ≤ 40 years, while 23 cases were above 40 years) without any Knee complaints (asymptomatic KOA).

Regarding the prevalence of KOA in relation to the gender of the participants, the prevalence of KOA among males was 27.7% (95% CI = 23.2 - 32.2%), while its prevalence among females was 75.6% (95% CI = 65.3 - 83.6%). The age and gender distributions of KOA-diagnosed cases are shown in Tables-II and III.

**Table II:** Age distribution of KOA.

Parameter		Total n (%)	KOA cases n (%)	KOA-free cases n (%)	P-value
Age groups	< 40 years	273 (100%)	70 (25.6%)	203 (74.4%)	<0.0001**** df = 72.53, 3
	40 – 50 years	75 (100%)	45 (60%)	30 (40%)	
	51 – 60 years	48 (100%)	38 (79.2%)	10 (20.8%)	
	61 – 90 years	14 (100%)	10 (71.4%)	4 (29.6%)	
	Total	410 (100%)	163 (39.7%)	247 (60.3%)	
Gender	Male	328 (100%)	102 (31%)	226 (69%)	<0.0001**** df=51.34, 1
	Female	82 (100%)	61 (74.4%)	21 (25.6%)	
	Total	410 (100%)	163 (39.7%)	247 (60.3%)	

**Table III:** Association between gender on and the prevalence of KOA in the different age groups.

Age group (years)	Males (Total) n (%)	Males with KOA n (%)	KOA-free males n (%)	Females (Total) n (%)	Females with KOA n (%)	KOA-free females n (%)	P-value
< 40	243 (100%)	56 (23%)	183 (77%)	30 (100%)	14 (46.7%)	16 (53.3%)	0.0063** df = 7.47, 1
40 – 50	49 (100%)	22 (44.9%)	26 (55.1%)	26 (100%)	23 (88.5%)	3 (11.5%)	0.0002*** df =12.86, 1
51 – 60	26 (100%)	17 (65.4%)	10 (34.6%)	22 (100%)	21 (95.5%)	1 (4.5%)	0.0067** df =7.35, 1
61 – 90	10 (100%)	7 (70%)	3 (30%)	4 (100%)	3 (75%)	1 (25%)	0.851 df = 0.58, 1
Total	328 (100%)	102 (31%)	226 (69%)	82 (100%)	61 (74.4%)	21 (25.6%)	

In the present study of MRI-diagnosed KOA, 91 (55.8%) cases were in the left side knees while 72 (44.2%) of participants showed KOA changes in the right side. Interestingly, there was no significant relationship between the side of the KOA and the age, gender or grades of OA in the study population.

MRI findings in cases with confirmed KOA diagnoses included cartilage injuries, osteophytes, meniscal tears, and bone marrow edemas. Cases with KOA were graded according to Park’s scoring system. Forty-four (26.9%) cases of KOA were categorized as Grade I, while 33 (20.2%) cases showed MRI criteria of Grade IIA KOA. The remaining 41 (25.2%) and 45 (27.6%) cases were determined as Grades IIB and III respectively according to their MRI findings. Additionally, 84% of Grade I KOA cases were below the age of 40 years old, while 91% of cases diagnosed as Grade III KOA were over 40 years old. The KOA grades by age and gender are shown in Table-IV.

**Table IV:** KOA grades in relation to ages and genders of the participant.

Group	Grade I n (%)	Grade IIA n (%)	Grade IIB n (%)	Grade III n (%)	P-value
<40 years	37 (84%)	20 (60.6%)	9 (21.9%)	4 (9%)	<0.0001**** df=63.28, 3
≥ 40 years	7 (16%)	13 (39.4%)	32 (78.1%)	41 (91%)	
Total	44 (100%)	33 (100%)	41 (100%)	45 (100%)	
Males	35 (79.5%)	24 (67.7%)	19 (46.3%)	24 (53.3%)	0.0044** df=13.12, 3
Females	9 (20.5)	9 (27.3%)	22 (53.7%)	21 (46.7%)	
Total	44 (100%)	33 (100%)	41 (100%)	45 (100%)	

## DISCUSSION

The current study has used MRI to investigate the prevalence of KOA in Saudi Arabia. The current data showed that the overall prevalence of general OA was about 39.7%. The prevalence of OA increased by age. However, KOA was also quite common among participants aged below 40 years (about 25% of enrolled participants aged below 40 years). KOA prevalence was higher in females than male participants. There was also a significant association between the participants’ age and gender and MRI-based grading.

The prevalence of OA was reported to be 13% in Alqaseem^21^ and 25.8% in Madinah^22^ through questionnaires and clinical examination studies, while OA prevalence was estimated to be around 56% using X-ray-based studies in Riyadh.^23^ The prevalence reported in these studies is different from ours due to differences in the studied population, year of the study, and the evaluation methods. In Asia, the Program for Control of Rheumatic Disorders (COPCORD) reported that OA prevalence ranges from 1.4% in urban Filipinos to 19.3% in the rural communities of Iran.^24^ A more recent 2016 study reported the prevalence of OA to be 28.7% in India.^25^ Internationally, the prevalence of OA in Canada was estimated to be 14.2% and in the United Kingdom among adults aged above 45 years old were around18.5%.^26^

The current study revealed OA prevalence in participants under 40 years old to be 25.6%. This prevalence increased along with age to reach 67.8% of the participants over 40 years old. This is in line with a previously published Saudi study conducted by Alamri et al. who revealed that 14% of patients with KOA were under 30 years old, 47% were 30 to 50 years old, and 39% were above the age of 50.^22^ Also, in a clinical examination-based study, Al-Arfaj et al. documented that the prevalence of OA was 30.8% in the age group of 46 to 55 year-olds and 60.6% in 66 to 75 year-olds.^23^ Other international publications also have also revealed this age-related difference in the prevalence of KOA.^24^-^26^ Additionally, in the present study, the grade of KOA was lower in younger patients compared to older patients. This positive correlation between grade and advancing age has been attributed to multiple factors such as the reduced response of chondrocytes to growth factors, higher concentrations of free radicals and abnormal accumulation of AGEs (advanced glycation end products).^27^

In the present study MRI diagnosed 30 (7.3%) cases of a symptomatic KOA. (7 cases ≤ 40 years, while 23 cases were above 40 years). These cases were about 10% of cases of KOA blow 40 and around 25% of KOA cases above 40 years. These percentages of asymptomatic KOA are within range reported by Culvenor et al.^28^, who reported that the prevalence of a symptomatic KOA diagnosed by MRI is 4%–14% in adults aged <40 years and 19%–43% in persons ≥40 years.

Bone marrow edema was shown in 53 cases of this study diagnosed KOA. All cases showed bone marrow lesions were symptomatic a cases which means that MRI showed edema in the bone marrow in 39.8% of the studied symptomatic cases. This is in accordance with Carotti et al.^29^ who reported that bone marrow lesions were found in 38.3% of MRI studied cases with symptomatic KOA.

Interestingly, the present study showed a higher prevalence among the adult population than what was published before in this age group. This rise in the incidence in younger age groups may be attributed to the increasing prevalence of obesity and the contribution of genetic factors, diet, and recreational activities.^30^ Alternatively, this rise may be due to the superior imaging and interpretation abilities afforded by MRI.

In addition, the current data showed a higher prevalence of KOA among the female relative to the male participants. This is in agreement with many previous studies.^21^-^26^ The reasons underlying the higher incidence of KOA among females may be due to various anatomical, genetic, and hormonal issues. Anatomically, females have narrower femurs, thinner patellae, larger quadriceps angles, and differences in tibial condylar size^31^, which could contribute to the development of KOA.

The findings of the current study are important for many reasons. Primarily, they revealed the relatively higher prevalence of KOA among the adult population in comparison with the previously reported results. Secondly, the current data highlight the importance of MRI as an effective, accurate tool for KOA diagnosis. Thirdly, the prevalence of KOA seems to be widely different in the different region of the kingdom, which supports the contribution of geographic and genetic factors to OA risk. Also the difference of the used assessment methods in the different studies significantly affected their overall results.

## Limitations of the study

The current study used MRI, for the first time in Saudi Arabia, to document the prevalence of OA. However it has some limitations. Firstly, Better MRI machine properties were required for better evaluation of the suggestive osteoarthritic lesions for better evaluation of cartilages defects, osteophytes, bone marrow edemas, and meniscal tears. However, the obtained data were sufficient for the grading system utilized in this study. Secondly, inspire the high ability of MRI to visualize the lesions and its progression in the different knee structure its sensitivity was estimated to be 61% (95% confidence interval [CI] 53 to 68), and specificity was 82% (95% CI 77 to 87) for diagnosis of osteoarthritis in the different joints, this does not underestimate the benefit of MRI as a diagnostic tool as even direct visualization of the lesion by arthroscopy of histology does not correlate perfectly with the clinical progression of the cases with KOA.^32^ Thirdly, limited number of female participants (only 20% of the participants) were enrolled in the current study, as most of relatives accompanied patients were males. In addition, most female cases, during the period of the study, did not accept to participate in the study; However the number of females is statistically accepted without so much effect on the final results and conclusions. Finally, other extended future studies should be conducted for better assessment of the OA problem burden all over Saudi Arabia for better planning to decrease OA-induced morbidity and disabilities.

## CONCLUSION

KOA is a common disease among the young population of Arar, especially among females. Its prevalence increases by age with higher grades of severity among elderly patients. Early management and patient education can alleviate KOA drawbacks. Further future studies are recommended to elaborate more about the factors affecting these results.

## References

[1] Neogi T (2013). The epidemiology and impact of pain in osteoarthritis. Osteoarthritis Cartilage.

[2] Palazzo C, Nguyen C, Lefevre-Colau MM, Rannou F, Poiraudeau S (2016). Risk factors and burden of osteoarthritis. Ann Physical Rehabil Med.

[3] Singer SP, Dammerer D, Krismer M, Liebensteiener MC (2018). Maximum lifetime body mass index is the appropriate predictor of knee and hip osteoarthritis. Arch Orthop Trauma Surg.

[4] Pal CP, Singh P, Chaturvedi S, Pruthi KK, Vij A (2016). epidemiology of knee osteoarthritis in India and related factors. Indian J Orthop.

[5] Plotnikoff R, Karunamuni N, Lytvyak E, Penfold C, Schopflocher D, Imayama I (2015). Osteoarthritis prevalence and modifiable factors:a population study. BMC Public Health.

[6] Liu B, Balkwill A, Green J, Beral V (2016). Body size from birth to middle age and the risk of hip and knee replacement. BMC Musculoskeletal Disord.

[7] Esch MVD, Knoop J, Leeden MVD, Roorda LD, Lems WF, Knol DL (2015). Clinical phenotypes in patients with knee osteoarthritis:a study in the Amsterdam osteoarthritis cohort. Osteoarthritis Cartilage.

[8] Litwic A, Edwards MH, Dennison EM, Cooper C (2013). Epidemiology and burden of osteoarthritis. Br Med Bull.

[9] Ginckel AV, Bennell KL, Campbell PK, Wrigley TV, Hunter DJ, Hinman RS (2016). Location of knee pain in medial knee osteoarthritis:patterns and associations with self-reported clinical symptoms. Osteoarthritis Cartilage.

[10] Mobasheri A, Batt M (2016). An update on the pathophysiology of osteoarthritis. Ann Physical Rehabil Med.

[11] Berenbaum F (2013). Osteoarthritis as an inflammatory disease (osteoarthritis is not osteoarthrosis!). Osteoarthritis Cartilage.

[12] Brandt K, Fife R, Raunstein E, Katz B (1989). Radiographic grading of the severity of knee osteoarthritis:relation of the Kellgren and Lawrence grade to a grade based on joint space narrowing and correlation with arthroscopic evidence of articular cartilage degeneration. Arthritis Rheum.

[13] Braun HJ, Gold GE (2012). Diagnosis of osteoarthritis:imaging. Bone.

[14] Harada Y, Tokuda O, Fukuda K, Shiraichi G, Motomura T, Kimura M (2011). Relationship between cartilage volume using MRI and Kellgren–Lawrence radiographic score in knee osteoarthritis with and without meniscal tears. Am J Roentgenol.

[15] Hayashi D, Roemer FW, Guermazi A (2016). Imaging for osteoarthritis. Ann Physical Rehabil Med.

[16] Schiphof D, Oei EHG, Hofman A, Waarsing JH, Weinans H, Bierma-Zeinstra SMA (2014). Sensitivity and associations with pain and body weight of an MRI definition of knee osteoarthritis compared with radiographic Kellgren and Lawrence criteria: a population-based study in middle-aged females. Osteoarthritis Cartilage.

[17] Joshi V, Singh R, Kohli N, Parashari U, Kumar A, Singh V (2008). Evaluation of osteoarthritis of the knee with magnetic resonance imaging and correlating it with radiological findings in the Indian population. Internet J Orthop Surg.

[18] Park HJ, Kim SS, Lee SY, Park NH, Park JY, Choi YJ (2013). A practical MRI grading system for osteoarthritis of the knee:association with Kellgren–Lawrence radiographic scores. Euro J Radiol.

[19] Noyes FR, Stabler CL (1989). A system for grading articular cartilage lesions at arthroscopy. Am J Sports Med.

[20] Newcombe RG (1998). Two-sided confidence intervals for the single proportion:comparison of seven methods. Stat Med.

[21] Al-Arfaj AS, Alballa SR, Al-Saleh SS, Al-Dalaan AM, Bahabry SA, Mousa MA (2003). Knee osteoarthritis in Al-Qaseem, Saudi Arabia. Saudi Med J.

[22] Alamri WM, Alamri AJ, Aljohani LZ, Almohammdi AF, Saber MS, Jamal R (2016). Prevalence and risk factors of osteoarthritis in Madinah, Saudi Arabia 2015. Int J Sci Res.

[23] Al-Arfaj A, Al-Boukai AA (2002). Prevalence of radiographic knee osteoarthritis in Saudi Arabia. Clin Rheumatol.

[24] Haq SA (2011). Osteoarthritis of the knees in the COPCORD world. Int J Rheum Dis.

[25] Pal CP, Singh P, Chaturvedi S, Pruthi KK, Vij A (2016). Epidemiology of knee osteoarthritis in India and related factors. Indian J Orthop.

[26] Birtwhistle R, Morkem R, Peat G, Williamson T, Green ME, Khan S (2015). Prevalence and management of osteoarthritis in primary care:an epidemiologic cohort study from the Canadian Primary Care Sentinel Surveillance Network. CMAJ Open.

[27] Li YP, Wei XC, Zhou JM, Wei L (2013). The Age-Related Changes in Cartilage and Osteoarthritis. Biomed Res Int.

[28] Culvenor AG, Oiestad BE, Hart HF, Stefanik JJ, Guermazi A, Crossley KM (2018). Prevalence of knee osteoarthritis features on magnetic resonance imaging in asymptomatic uninjured adults:a systematic review and meta-analysis. Br J Sports Med.

[29] Carotti M, Salaffi F, Di Carlo M, Giovagnoni A (2017). Relationship between magnetic resonance imaging findings, radiological grading, psychological distress and pain in patients with symptomatic knee osteoarthritis. La Radiologia Medica.

[30] Amoako AO, Pujalte GGA (2014). Osteoarthritis in Young, Active, and Athletic Individuals. Clin Med Insights Arthritis Musculoskelet Disord.

[31] Hame SL, Alexander RA (2013). Knee osteoarthritis in women. Curr Rev Musculoskeletal Med.

[32] Menashe L, Hirko K, Losina E, Kloppenburg M, Zhang W, Li L, Hunter DJ (2012). The diagnostic performance of MRI in osteoarthritis:a systematic review and meta-analysis. Osteoarthritis Cartilage.

